# The effect of cognitive task on ankle movement variability in athletes with Functional Ankle Instability

**DOI:** 10.1186/1757-1146-7-S1-A90

**Published:** 2014-04-08

**Authors:** Sanam Tavakoli, Saeed Forghany, Christopher Nester, Akram Jamali, Khadijeh Bapirzadeh

**Affiliations:** 1 Musculoskeletal Research Centre, Isfahan University of Medical Sciences, Iran; 2 Centre for Health Sciences Research, University of Salford, UK

## Background

Gait has been generally viewed as a largely automated motor task, requiring minimal higher-level cognitive input. Increasing evidence, however, suggest that attention demanding cognitive tasks to disturb gait [1,2]. Movement variability may influence joint stability and increase the risk of “giving way” at the ankle in individuals with functional ankle instability (FAI) [3]. The purpose of this study was to investigate the effect of dual-tasking on ankle movement variability in athletes with FAI.

## Methods

21 athletes (age 25.57±4.77 years) with clinically diagnosed FAI were recruited. All participants completed 5 trials of normal walking and 5 trials of normal walking while performing a cognitive task. The cognitive task consisted of subtracting seven from a randomly selected number between 11 and 99 repeatedly whilst walking. Three dimensional rotations of the affected ankle (measured by an eight-camera motion capture system at 100 Hz) were calculated by visual3D during gait cycles. Between trials variability of ankle rotations time curves during stance phase and during 200ms before and after heel strike were calculated using the coefficient of multiple correlations (CMC) and intraclass correlation (ICC)

## Results

The results indicate that mean CMC was decreased during dual task condition in the sagittal and frontal planes. This was statistically significant in frontal plane during 200ms before and after heel strike (p<0.05) (Table 1). There was reduction in ICC magnitude in dual-task condition compared to single task in 200ms before heel strike (Table 2).

**Table 1:** Mean CMC during different conditions and periods of time.

		Single-Task	Dual-Task
200ms before and after HS^a^	Frontal plane	0.9529±0.029	0.9270±0.044 *
	
	Sagittal plane	0.9505±0.042	0.9373±0.046
	
	Transverse plane	0.8530±0.150	0.8539±0.140

HS-TO^b^	Frontal plane	0.9396±0.042	0.91150.092
	
	Sagittal plane	0.9842±0.019	0.9825±0.022
	
	Transverse plane	0.9228±0.092	0.9274±0.072

^a^ Heel strike. ^b^ Toe off. ^*^ P <0.05

**Table 2:** ICC in 3planes during different conditions

		Single-Task	Dual-Task
200ms before HS ^a^	Frontal plane	0.964	0.960
	
	Sagittal plane	0.943	0.710
	
	Transverse plane	0.934	0.914

HS	Frontal plane	0.968	0.975
	
	Sagittal plane	0.879	0.907
	
	Transverse plane	0.756	0.908

200ms after HS	Frontal plane	0.958	0.909
	
	Sagittal plane	0.950	0.949
	
	Transverse plane	0.809	0.973

TO ^b^	Frontal plane	0.911	0.930
	
	Sagittal plane	0.882	0.898
	
	Transverse plane	0.924	0.903

^a^ Heel strike. ^b^ Toe off. ^*^ P <0.05

## Conclusion

The athletes with FAI demonstrated greater ankle movement variability during dual task condition which may indicate diminished neuromotor control. Cognitive load may increase episodes of ankle instability in these athletes.

## Competing interests

Nester declares a personal commercial interest in the insoles tested in this study.
